# Screening for cervical cancer: a systematic review and meta-analysis

**DOI:** 10.1186/2046-4053-2-35

**Published:** 2013-05-24

**Authors:** Leslea Peirson, Donna Fitzpatrick-Lewis, Donna Ciliska, Rachel Warren

**Affiliations:** 1 McMaster Evidence Review and Synthesis Centre (MERSC), 1280 Main Street West, DTC-322, Hamilton, ON L8S 4K1, Canada

**Keywords:** Cervical cancer, Screening, Systematic review

## Abstract

**Background:**

The systematic review on which this paper is based provided evidence for the Canadian Task Force on Preventive Health Care to update their guideline regarding screening for cervical cancer. In this article we highlight three questions covered in the full review that pertain to the effectiveness of screening for reducing cervical cancer mortality and incidence as well as optimal timing and frequency of screening.

**Methods:**

We searched MEDLINE, Embase and Cochrane Central from 1995 to 2012 for relevant randomized controlled trials and observational studies with comparison groups. Eligible studies included women aged 15 to 70 years who were screened using conventional cytology, liquid-based cytology or human papillomavirus DNA tests. Relevance screening, data extraction, risk of bias analyses and quality assessments were performed in duplicate. We conducted a meta-analysis using a random-effects model on the one body of evidence that could be pooled.

**Results:**

From the 15,145 screened citations, 27 papers (24 studies) were included; five older studies located in a United States Preventive Services Task Force review were also included. A randomized controlled trial in India showed even a single lifetime screening test significantly decreased the risk of mortality from and incidence of advanced cervical cancer compared to no screening (mortality: risk ratio 0.65, 95% confidence interval 0.47, 0.90; incidence: relative risk 0.56, 95% confidence interval 0.42, 0.75). Cytology screening was shown to be beneficial in a cohort study that found testing significantly reduced the risk of being diagnosed with invasive cervical cancer compared to no screening (risk ratio 0.38; 95% confidence interval 0.23, 0.63). Pooled evidence from a dozen case–control studies also indicated a significant protective effect of cytology screening (odds ratio 0.35; 95% confidence interval 0.30, 0.41). This review found no conclusive evidence for establishing optimal ages to start and stop cervical screening, or to determine how often to screen; however the available data suggests substantial protective effects for screening women 30 years and older and for intervals of up to five years.

**Conclusions:**

The available evidence supports the conclusion that cervical screening does offer protective benefits and is associated with a reduction in the incidence of invasive cervical cancer and cervical cancer mortality.

## Background

Cervical cancer is a treatable disease and tertiary interventions have contributed to reductions in mortality rates [1]. However, when downstream activities are combined with preventive efforts, there is greater impact in terms of lives saved. There is widespread acceptance that regular screening is the single most important public health strategy to reduce cervical cancer incidence and subsequent mortality. Screening tests such as conventional cytology (commonly referred to as the Pap smear) are used to identify pre-cancers, which can be treated to prevent the occurrence of invasive cancer or allow the disease to be identified at an earlier stage, permitting more effective treatment. The systematic review on which this paper is based provided evidence for the Canadian Task Force on Preventive Health Care to update their guideline regarding screening of average-risk women for cervical cancer [2,3].

Much of the recent research has focused on reductions in precursor cervical lesions, which are more common and present earlier outcomes for measurement during trials. However, evidence from a treated versus not-treated cohort study with 30-year follow-up suggests a third of cases with precursor lesions will advance to invasive cancer [4]. Consequently, cervical cancer mortality and incidence of invasive cervical cancer were designated as the critical outcomes for this review. To our knowledge, no published systematic review has addressed the question of screening effectiveness with these outcomes, and until recently there were no randomized controlled trials (RCTs) to inform decision-making. Instead, ecological studies have been used to demonstrate that mortality from cervical cancer decreased as screening became more widespread [5,6]. However, higher level evidence is available. In this article we highlight three questions covered in the full systematic review:

1. What is the effect of cervical cancer screening on incidence of and mortality from invasive cervical cancer?

2. How does varying the screening interval affect incidence of and mortality from invasive cervical cancer?

3. How does varying the age at which screening is started or stopped reduce incidence of and mortality from invasive cervical cancer?

The PICOS (population, intervention, comparator, outcome, study design) framework for these questions was as follows: (P) asymptomatic women aged 15 to 70 years with a history of sexual activity, (I) conventional cervical cytology, liquid-based cervical cytology or human papillomavirus (HPV) DNA screening tests, (C) no screening, (O) cervical cancer mortality and incidence of invasive cervical cancer, and (S) RCTs and observational studies with comparison groups.

## Methods

### Search strategy and eligibility criteria

For the questions addressed in this paper, MEDLINE, Embase and Cochrane Central Register of Controlled Trials were searched from 1995 to April 2012 for studies conducted in any country and published in English or French. As above, results were limited to systematic reviews, RCTs and observational studies with comparison groups, involving asymptomatic women aged 15 to 70 years with a history of sexual activity, who were screened using conventional cytology, liquid-based cytology or HPV DNA tests. These specific tests were purposefully selected as they are the most relevant screening modalities for the Canadian context, for which subsequent screening guidelines would be developed. The outcomes of interest were cervical cancer mortality and incidence of invasive cervical cancer. In addition, we examined two United States Preventive Services Task Force (USPSTF) reviews on cervical screening for any relevant studies not captured by our search strategy [7,8]. Reference lists of on-topic systematic reviews were also searched to ensure all primary studies meeting our inclusion criteria were considered. A focused search of PubMed, specifically for RCTs on cervical screening, was undertaken on November 5 2012, several weeks in advance of the release of the Canadian Task Force on Preventive Health Care guideline.

### Study selection and data extraction

Pairs of reviewers screened all identified citations. Any citation deemed potentially relevant was retrieved for full text review. Two reviewers independently assessed each full text article for eligibility. Disagreements were resolved through discussion. For each included study, two reviewers independently extracted relevant outcome data and study details. Conflicts were resolved through discussion.

### Quality assessment

Risk of bias assessments were completed in duplicate. Quality of the evidence was determined using the GRADE system (Grading of Recommendations Assessment, Development and Evaluation). This considers five criteria (design, consistency, directness, precision, reporting bias) to rate of a body of evidence as high, moderate, low or very low, indicating the assessment of the likelihood that further research will impact the estimate of effect [9]. After two reviewers independently assessed the evidence on the criteria, agreement was reached on the ratings and the overall quality of the summary statistics.

### Statistical analysis

For the one included RCT we combined the cytology and HPV testing arms into a single screening group that was compared to the control group on the outcomes of cervical cancer mortality, incidence of all cervical cancer, and incidence of stage II or higher cervical cancer. Event rates were entered into Cochrane’s Review Manager 5 (RevMan) software [10] and a fixed-effects model was used to compute a risk ratio for each outcome.

A single cohort study provided data for the incidence outcome. To be consistent in the presentation of findings, the reported estimate of effect was inverted to provide the risk for the screened group with the unscreened group as the referent.

We also conducted a meta-analysis using data from case–control studies that examined the odds of exposure to cytology screening among women diagnosed with invasive cervical cancer and women with no history of the disease. RevMan [10] was used to perform the meta-analysis using the generic inverse variance method and the random-effects model [11]. We used Chi^2^ and I^2^ values to test for heterogeneity [12-14]. Post hoc sensitivity analyses (based on contextually and clinically important differences in study designs, populations, interventions, organized versus opportunistic approaches, and length of exposure to screening) were conducted to attempt to explain heterogeneity.

## Results

### Study selection and characteristics

**Figure 1:**
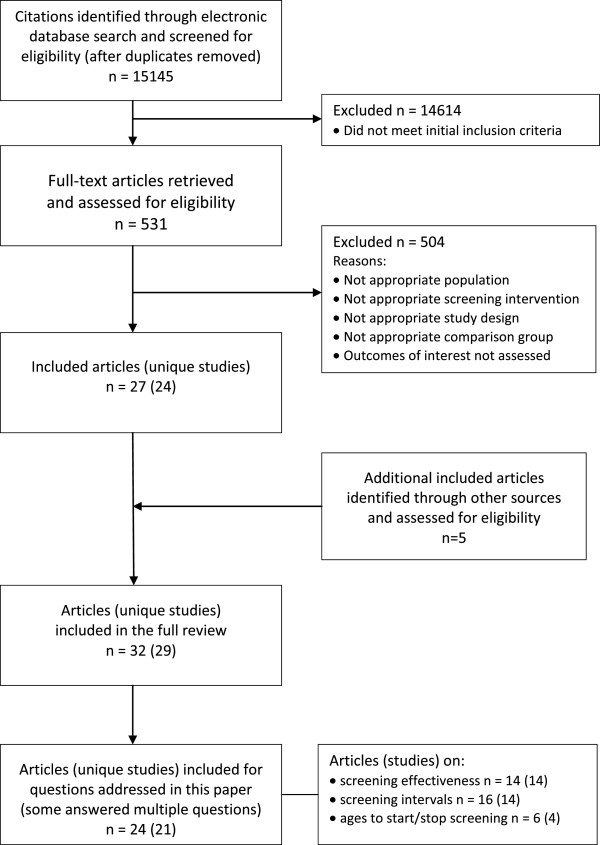
Flow diagram for selection of studies included in the systematic review.

Figure 1 shows the selection of studies. The search strategy located 15,145 citations after duplicates were removed, approximately 4% (n = 531) of which passed relevance screening and went on to full text assessment. Of these, 27 papers (24 unique studies) met all criteria and were included in the full review. In addition, five studies in the 1996 USPSTF report that pre-dated our search parameters were added to the evidence [15-19]. The 2011 USPSTF report contained no studies that met our inclusion criteria that were not already part of this review and no additional primary studies were located in the reference lists of other systematic reviews. The supplemental search of PubMed in November 2012 located 10 new citations, however none of these were RCTs that met the inclusion criteria for this review.

For the three questions covered in this paper, we identified one RCT [20], two cohort studies [21,22] and 18 case–control studies (one study had four publications) [15-19,23-38]. Fourteen of these studies were used to examine the question of screening effectiveness, 14 studies (16 papers) provided data on screening intervals, and four studies (six papers) provided data that considered ages to initiate and discontinue screening. The RCT considered both cytology and HPV screening tests; all observational studies considered only cytology. Additional characteristics of these 21 studies are summarized in Table 1.

**Table 1:** Characteristics of the included studies

**Randomized (cluster) controlled trial**									
**Study**	**Location**	**Number of participants**		**Ages (years)**	**Screening exposure**	**Disease definition**	**Participant eligibility**	**Post screening treatment**	**Follow-up**
		**Intervention**	**Control**						
Sankaranarayanan *et al*. [20]	India	HPV: 34,126 Cytology: 32,058	31,488	30 to 59	Only 8 (<0.007%) of the eligible women had ever been screened	Invasive cervical cancer; FIGO stages I+	No history of cervical cancer	Cryotherapy, LEEP, conization offered for CIN; invasive cancer referred for treatment (surgery, radiotherapy)	8 years 2000 to 2007
**Cohort studies**									
**Study**	**Location**	**Number of participants**		**Ages (years)**	**Screening exposure**	**Disease definition**	**Participant eligibility**	**Post screening treatment**	**Follow-up**
Herbert *et al.*[21]	UK	116,022 (four groups based on screening history)		25 to 69	Interval since last smear: <3.5 y, 3.5 to 5.5 y, >5.5 y, no record	Invasive cervical cancer; FIGO stages I+	No hysterectomy	NR	3 years 1991 to 1993
					Excluded: smears to investigate symptoms, referral smears in screen-detected cases				
Rebolj *et al*. [22]	Netherlands	Cohort 1: 445,382 Cohort 2: 218,847		C1: 30 to 44 C2: 45 to 54	Mean interval between three consecutive negative results	Invasive cervical cancer; FIGO stages NR	Three consecutive negative smears in age interval; no history of CIN or cytological abnormalities	NR	10 years 1994 to 2002
					C1: 39 m				
					C2: 40 m				
**Case control studies**									
**Study**	**Location**	**Number of participants**		**Ages (years)**	**Screening exposure/history**	**Case/disease definition**	**Control eligibility**	**Post screening treatment**	**Case diagnosis dates**
		**Cases**	**Controls**						
Andrae *et al.*[24]	Sweden	1,230	6,124	20 to 99	Interval since last smear: 6 to 42 m (ages <53), 6 to 66 m (ages 54 to 65), 6 to 78 m (ages ≥66), not screened during interval	Invasive cervical cancer; FIGO stages 1+	No history of cervical cancer; alive on case diagnosis date	NR	1999 to 2001
					Excluded: smears <6 m prior to case diagnosis				
Aristizabal *et al*. [15]	Colombia	277	554	16 to 60	Interval since last smear: 12 to 72 m prior to case diagnosis	Invasive cervical cancer; excluded *in situ*	Out-patient services from same clinic where case diagnosed or reside in same area as case	NR	1977 to 1981
					Excluded: smears <12 m prior to diagnosis/index date				
Berrino *et al.*[16]	Italy	121	350	NR	Interval since last negative smear: 0 to 11 m, 12 to 23 m, 24 to 35 m, 36 to 47 m, 48+ m, no prior negative smear	Invasive cervical cancer; FIGO stages I+	Married women hospitalized for and diagnosed with non-gynecological diseases; no history of breast cancer or hysterectomy	Two controls had been treated for carcinoma *in situ* after a positive smear result	1978
					Included: smears prior to symptoms (cases), smears prior to study mid-point (controls)				
Clarke and Anderson [17]	Canada	212	1,060	Mean 52	Interval since last smear: <5 y prior to case diagnosis	Invasive cervical cancer; most <6 m post diagnosis	No exclusion for hysterectomy	NR	1973 to 1976
					Included: non-symptomatic, routine examination smears				
Decker *et al.*[25]	Canada	666	3,343	≥18 Mean 50	Interval since last smear: <5 y prior to case diagnosis	Invasive cervical cancer; FIGO stages I+	No history of cervical cancer or malignant neoplasms (excluding non-melanoma skin cancer); no hysterectomy	NR	1989 to 2001
					Excluded: smears <6 m prior to case diagnosis				
Hernández-Avila *et al*. [26]	Mexico	397	1,005	25 to 80 Mean 48	Exposure: any lifetime smear(s), no history	Invasive cervical cancer	Eligibility limited only by age and area of residence	NR	1990 to 1992
					Excluded: smears <12 m prior to case diagnosis or control interview				
Herrero *et al.*[18]	Latin America	759	1,430	<70	Interval since last smear: 12 to 23 m, 24 to 47 m, never screened	Invasive cervical cancer; FIGO stages I+	No history of psychiatric diagnoses, hysterectomy, cancer, or diseases related to exposures of interest; history of sexual intercourse	NR	1986 to 1987
					Excluded: smears <1 y prior to interview (mean interval between case diagnosis and: case interview 1 m; control interview 2.3 m)				
Hoffman *et al.*[27]	South Africa	524	1,540	<60	Interval since last smear: <5 y, 5 to 9 y, 10 to 14 y, ≥15 y, unknown, never	Invasive cervical cancer; FIGO stages IB to IV; diagnosed ≤6 m prior to study enrollment	Hospital admission not related to risk of cervical cancer	NR	NR late 1990s
					Analysis excluding smears in prior 12 m showed no difference in findings				
Jiménez-Pérez and Thomas [28]	Mexico	143	311	Mean 49	Interval since last smear: 1 to 12 m, 13 to 60 m, >60 m, unknown, never	Invasive cervical cancer; FIGO stages IB to IV	No hysterectomy; not attending clinic for cervical screening, or any gynecologic or obstetric conditions; history of sexual intercourse	NR	1991 to 1994
					Excluded: smears ≤12 m prior to diagnosis/index date				
Kasinpila *et al.*[38]	Thailand	130	260	Mean 48	Interval since last smear: <6 m, 6 to 11 m, 12 to 35 m, 36+ m, never	Invasive cervical cancer; diagnosed ≤3 m prior to interview	No evidence of cervical disease or any other gynecological disease	Cryotherapy and LEEP offered for confirmed abnormalities	2009
					Excluded: smears in previous 6 m				
La Vecchia *et al.*[19]	Italy	191	191	22 to 74	Interval since last smear: <3 y, 3 to 5 y, >5 y, never	Invasive cervical cancer; FIGO Stages 1+	Admitted and diagnosed with acute non-malignant, non-hormonal, non-gynecological problems <1 y prior to interview	NR	1981 to 1983
					Excluded: smears to investigate symptoms; cases with positive smear <1 y prior to diagnosis				
Makino *et al*. [29]	Japan	129	396	35 to 79	Interval since last negative smear to diagnosis/index date: 1 y, 2 y, 3 y, 4 y, ≥5 y	Invasive cervical cancer; diagnosed <6 m after abnormal smear result	No invasive cervical cancer; no hysterectomy; no previous abnormal cytology results; participated in mass screening	NR	1984 to 1990
					Exposure: any lifetime smear(s), no history				
					Excluded: smears to investigate symptoms or taken at time of diagnosis				
Miller *et al.*[31]	USA	482	934	Mean 49	Interval from last negative smear to diagnosis/index date: 1 y (0 to 18 m), 2 y (19 to 30 m), 3 y (31 to 42 m), 3 to 5 y (42 to 66 m), 5 to 10 y (67 to 126 m), >10 y (>126 m)	Invasive squamous cell cervical cancer	No prior hysterectomy or radiation to the pelvis	NR	1983 to 1995
Nieminen *et al.*[23]	Finland	179	1,507	30 to 91 Mean 60	Exposure: any lifetime smear(s), no history	Invasive cervical cancer	Eligibility limited only by catchment (reside in area served by case treatment hospital)	Organized screening program provides colposcopy examinations and treatment for mild abnormalities	1987 to 1994
					Excluded: smears <1 y prior to diagnosis/index date				
Sasieni *et al.*[32]	UK	348	677	≥20	Interval since last negative smear (not immediately following a test showing abnormal results): 0 to 11 m, 12 to 23 m, 24 to 35 m, 36 to 47 m, 48 to 65 m, >66 m, no history	Invasive cervical cancer; FIGO stages IB+	No hysterectomy	NR	1992
Sasieni *et al.*[33]	UK	1,305	2,532	20 to 69	Interval since last negative smear (no abnormal smear in prior 12 m): <3 y, 3<5 y, 5+ y, no history of a negative smear	Invasive cervical cancer; FIGO stages IB+	No hysterectomy	NR	1990 to 2001
Sasieni *et al.*[34]	UK	4,012	7,889	20 to 69	Exposure: screening or no screening during specific age bands (for example, 20 to 21, 22 to 24, 20 to 24) related to cancers diagnosed in specific and imminent age bands (for example, 25 to 29)	Invasive cervical cancer; FIGO stages I+	No hysterectomy; registered with a National Health Services general practitioner, still alive, not emigrated	NR	1990 to 2008
Sasieni *et al.*[35]	UK	3,305	6,516	20 to 69	Maximum interval between smears: <3.5 y, 3.5 to 5.5 y, >5.5 y or no smear history	Invasive cervical cancer; FIGO stages I+	No hysterectomy	NR	1990 to 2008
Talbott *et al.*[30]	USA	143	143	Mean 45	Exposure: smear <3 y prior to case diagnosis or control interview	Invasive cervical cancer (localized, regional or distant); excluded *in situ*	No hysterectomy	NR	1984 to 1985
					Diagnostic smears: any positive result <12 m prior to diagnosis				
Yang *et al.*[36]	Australia	877	2,614	20 to 69	Exposure: 0, 1 or 2+ smears in last 4 y	Invasive cervical cancer (localized and non-localized)	No invasive cervical cancer diagnosis 1996 to 2003; alive at case diagnosis; no hysterectomy before end point	NR	2000 to 2003
					Excluded: smears <3 m prior to case diagnosis				
Zappa *et al.*[37]	Italy	208	832	<70	Interval since last smear (prior to index date): ≤3 y, 3 to 6 y, 6+ y, no record	Fully invasive cervical cancer; excluded micro-invasive	No hysterectomy prior to index date; alive at index date	NR	1994 to 1999
					Excluded: smears <12 m prior to diagnosis/index date				

CIN, Cervical intraepithelial neoplasia; FIGO, International Federation of Gynecology and Obstetrics; LEEP, loop electrosurgical excision procedure; NR, not reported; m, months; y, years.

### Quality assessment

Despite concerns regarding sample selection, the one included RCT [20] was otherwise judged to have low risk of bias (Table 2). However, this research was downgraded to a moderate quality GRADE rating due to serious concerns about indirectness of the evidence for this review (Table 3). Only one of the included cohort studies [21] had exposed and unexposed groups and could therefore be assessed for risk of bias with the selected scale [39]. This study satisfied all of the criteria except one and was given a low risk rating (Table 4). Though not downgraded for any serious concerns, this evidence received an overall low quality GRADE rating (Table 3). Based on the rationale that observational research involves less rigorous methods than RCTs, these types of studies start with a low quality rating in the GRADE system [9]. Overall the 18 included case–control studies demonstrated low risk of bias (Table 4). All but two studies scored six or more out of a possible nine points on the scale with a median rating of seven. The two most common risks were use of hospital controls (eight studies) and use of self-reports and/or non-blinded interviews to ascertain exposure (nine studies). As a group, the 12 case–control studies used to answer the effectiveness of screening question was downgraded to very low GRADE quality due to concerns about indirectness of the body of evidence to the Canadian context as well as the strong likelihood of publication bias (Table 3). It should also be noted that half of these papers contained data that are at least 20 years old and all were based on screening that occurred more than 10 years ago, prior to the introduction of HPV testing.

**Table 2:** Risk of bias assessment of the randomized controlled trial

**Study**	**Adequate sequence generation**	**Concealment of allocation**	**Blinding of participants and personnel**	**Blinding of outcome assessment**	**Incomplete outcome data**	**Selective reporting**	**Other bias**
Sankaranarayanan *et al*. [20]	Unclear: Does not specify	High risk: Probably not done	Low risk: Not possible; unlikely to influence results	Low risk: Probably done	Low risk: Analysis by intention to screen	Low risk: All outcomes of interest reported	Low risk: No other sources of bias observed

Risk of bias was assessed using the Cochrane Risk of Bias Tool [40].

**Table 3:** Summary of findings for effect of screening on cervical cancer mortality and incidence

**Outcome**	**Illustrative comparative risks**^**a**^				
	**Assumed risk for no screening Number per million**	**Corresponding risk for screening Number per million (95% CI)**	**Relative effect (95% CI)**	**Number of participants (Number of studies)**	**GRADE quality of evidence**^**b**^
Cervical cancer mortality (invited to HPV test or cytology versus no screening) RCT; follow-up: 8 years	2,033^c^	1,330 (964, 1,834)^c^	RR 0.65 (0.47, 0.90)^d^	97,672 (1^e^)	Moderate^f,g,h,i,j^
Incidence of stage II+ cervical cancer (invited to HPV test or cytology versus no screening) RCT; follow-up: 8 years	2,604^c^	1,466 (1,093, 1,966)^c^	rr 0.56 (0.42, 0.75)^d^	97,672 (1^e^)	Moderate^f,g,h,i,j^
Incidence of invasive cervical cancer (invited to HVP test or cytology versus no screening) RCT; follow-up: 8 years	3,747^c^	4,216 (3,401, 5,226)^c^	rr 1.12 (0.91, 1.39)^d^	97,672 (1^e^)	Moderate^f,g,h,i,j^
Incidence of invasive cervical cancer (cytology versus no screening) cohort study; follow-up: 3 years	1,596^k^	609 (368, 1,006)^l^	rr 0.38 (0.23, 0.63)	116,022 (1^m^)	Low ^g,i,j,n^
Exposure to cytology screening (cases: diagnosed with invasive cervical cancer; controls: no cervical cancer); exposure: in previous 3 years to lifetime	4,781 cases and 17,916 controls		OR 0.35 (0.30, 0.41)	22,697 (13^o^)	Very low ^p,q,r,s^

^a^ The assumed risk is the median control group risk. The corresponding risk (and its 95% CI) is based on the assumed risk in the comparison group and the relative effect of the intervention (and its 95% CI). ^b^ High quality: Further research is very unlikely to change our confidence in the estimate of effect; Moderate quality: Further research is likely to have an important impact on our confidence in the estimate of effect and may change the estimate; Low quality: Further research is very likely to have an important impact on our confidence in the estimate of effect and is likely to change the estimate; Very low quality: We are very uncertain about the estimate. ^c^ Rates were adjusted for age by study authors. ^d^ Study authors do not provide a hazard ratio for the HPV testing and cytology groups combined versus the control group. Using sample and event data we computed a risk ratio/relative risk. ^e^ Sankaranarayanan *et al*. [20]. ^f^ Random sequence generation unclear and allocation concealment not described, however study limitations were not downgraded for these risks/uncertainties. ^g^ Single study, therefore inconsistency not applicable. ^h^ Directness downgraded due to concerns regarding generalizability of population characteristics (rural women living in a low-income country) and intervention characteristics (one-time opportunistic screening; short duration (3 months) of training received by laboratory technicians responsible for processing and reading the samples) to Canadian context. ^i^ The number of events is small (<300, a threshold rule of thumb value for dichotomous outcomes), however considering the specific outcome the evidence is not downgraded. ^j^ Insufficient number of studies to assess publication bias [41]. ^k^ Twenty cases of cervical cancer diagnosed in women who had been screened in the 0.5- to 5.5-year interval; six of these cases were screen-detected cancers while 14 cases were symptomatic cancers. ^l^ Sixty-three cases of cervical cancer diagnosed in women who had been screened in the 0.5- to 5.5-year interval; 37 of these cases were screen-detected cancers while 26 cases were symptomatic cancers. ^m^ Herbert *et al*. [21]. ^n^ Newcastle-Ottawa Scale [39] for cohort studies was completed to assess study limitations; eight out of a possible nine stars were awarded. ^o^ There are 12 included case–control studies [15-18,23-30]. The number of studies appears as 13 because two different data sets from one study [23] were used as separate entries in the meta-analysis. ^p^ Newcastle-Ottawa Scale [39] completed to assess study quality. None of the studies satisfied all of the rating criteria. Despite some uncertainties (for example, lack of information on non-response rates in some studies) and limitations (for example, one-third of the studies used hospital controls rather than community controls), the evidence was not downgraded for study limitations. ^q^ The CIs overlap and the directness of the effect is consistent across studies (see Figure 2); all studies favor screening and only two of the 13 CIs marginally intersect the line of no difference. However, statistical heterogeneity is high (Chi^2^ = 50.98, df = 12 (*P* <0.00001); I^2^ = 76%). Sensitivity analyses were conducted and moderate heterogeneity was found when testing for differences between studies conducted in generalizable (to Canadian context) countries versus less generalizable countries (Chi^2^ = 2.27, df = 1, *P* = 0.13, I^2^ = 55.95) but minimal to no heterogeneity (I^2^ 0% to 21.6%) was found when other differences were explored (that is, Canada and US versus other countries, screening approaches, recency of exposure, sources of screening history, diagnosis dates, sources of controls). ^r^ Directness downgraded due to concerns regarding inclusion of both organized and opportunistic screening approaches; diversity of study locations which included developed and developing countries (Canada, US, Finland, Sweden, Japan, Italy, South Africa, Columbia, Costa Rica, Panama, Mexico); and the related potential for important differences in participants and screening procedures, particularly given that half of the studies looked at screening that occurred more than 20 years ago and all studies looked at screening that occurred more than 10 years ago. ^s^ Publication bias was strongly suspected due to asymmetry in the funnel plot and the recognition that the risk of publication bias may be substantial for observational studies, particularly small studies that utilize data from electronic medical records or disease registries [41,42]. CI, confidence interval; GRADE, Grading of Recommendations Assessment, Development and Evaluation; HPV, human papillomavirus; OR, odds ratio; RCT, randomized controlled trial; rr, relative risk; RR, risk ratio.

**Table 4:** **Risk of bias assessment of the observational studies**^**a**^

**Cohort study**	**Representativeness of exposed cohort**	**Selection of unexposed cohort**	**Ascertainment of exposure**	**Demonstration that outcome not present at study start**	**Comparability of cohorts on age**	**Comparability of cohorts on other factors**	**Assessment of outcome**	**Adequate length of follow-up**	**Adequacy of follow-up cohorts**	**Overall score**^**b**^
Herbert *et al. *[21]	✓ Truly representative	✓ Same community	✓ Secure record	✓ Yes	✓ Yes	No	✓ Record linkage	✓ Yes (3 years)	✓ All subjects followed	8
Rebolj *et al. *[22]	✗									
**Case control study**	**Adequate case definition**	**Representative cases**	**Selection of controls**	**Definition of controls**	**Comparability of controls on age**	**Comparability of controls on other factors**	**Ascertainment of exposure**	**Method of ascertainment**	**Non-response rate**	**Overall score**^**b**^
Andrae *et al. *[24]	✓ Independently validated	✓ Consecutive cases	✓ Community controls	✓ No history of disease	✓ Yes	No	✓ Secure record	✓ Same for both groups	✓ Same for both groups	8
Aristizabal *et al.*[15]	✓ Independently validated	✓ Representative cases	✓ Community and hospital controls	Not stated	✓ Yes	✓ Neighborhood	✓ Secure record and non-blinded interview	✓ Same for both groups	Not stated	7
Berrino *et al.*[16]	✓ Independently validated	✓ Consecutive cases	Hospital controls	✓ No history of disease	✓ Yes	No	✓ Secure record	✓ Same for both groups	✓ Same for both groups	7
Clarke and Anderson [17]	✓ Independently validated	✓ Representative cases	✓ Community controls	Not stated	✓ Yes	✓ Neighborhood and type of dwelling	✓ Secure record and non-blinded interview	✓ Same for both groups	Non-respondents described	7
Decker *et al.*[25]	✓ Independently validated	✓ Consecutive cases	✓ Community controls	✓ No history of disease	✓	✓ Area of residence	✓ Secure record	✓ Same for both groups	✓ Same for both groups	9
Hernández-Avila *et al.*[26]	✓ Independently validated	✓ Representative cases	✓ Community controls	Not stated	✓Yes	✓ Age of sexual debut, # normal births, # sex partners, SES	Non-blinded interview	✓ Same for both groups	Rate different/no designation	6
Herrero *et al.*[18]	✓ Independently validated	Not stated	Hospital controls	✓ No history of disease	No	No	Non-blinded interview	✓ Same for both groups	✓ Same for both groups	4
Hoffman *et al.*[27]	✓ Independently validated	Not stated	Hospital controls	✓ No history of disease	✓ Yes	✓ Race, area of residence, hospital	Interview	✓ Same for both groups	✓ Same for both groups	6
Jiménez-Pérez and Thomas [28]	✓ Independently validated	✓ Consecutive cases	Hospital controls	✓ No history of disease	✓ Yes	✓ Area of residence	Non-blinded interview	✓ Same for both groups	✓ Same for both groups	7
Kasinpila *et al.*[38]	✓ Independently validated	✓ Consecutive cases	Hospital controls	✓ No history of disease	✓ Yes	✓ Significant risk factors	Non-blinded interview	✓ Same for both groups	✓ Same for both groups	7
La Vecchia *et al.*[19]	✓ Independently validated	✓ Representative cases	Hospital controls	✓ No history of disease	✓ Yes	No	Interview	✓ Same for both groups	✓ Same for both groups	6
Makino *et al.*[29]	✓ Independently validated	Potential for selection bias	✓ Community controls	✓ No history of disease	✓ Yes	✓ Area of residence	Self-report	✓ Same for both groups	✓ Same for both groups	7
Miller *et al.*[31]	✓ Independently validated	Potential for selection bias	Hospital controls	✓ No history of disease	✓ Yes	✓ Length of membership in health program, race/ethnicity	✓ Secure record	✓ Same for both groups	✓ Same for both groups	7
Nieminen *et al.*[23]	✓ Independently validated	✓ Consecutive cases	✓ Community controls	Not stated	✓ Yes	✓ Socio-demographics, parity, smoking	Self-report	✓ Same for both groups	Rate different/no designation	6
Sasieni *et al.*[32-35]	✓ Independently validated	✓ Consecutive cases	✓ Community controls	Not stated	✓ Yes	✓ Area of residence	✓ Secure record	✓ Same for both groups	✓ Same for both groups	8
Talbott *et al.*[30]	✓ Independently validated	✓ Consecutive cases	✓ Community controls	Not stated	✓ Yes	✓ Sex, race, street or neighborhood	Non-blinded interview	✓ Same for both groups	✓ Same for both groups	7
Yang *et al.*[36]	Record linkage	Not stated	Hospital controls	✓ No history of disease	✓ Yes	No	✓ Secure record	✓ Same for both groups	✓ Same for both groups	5
Zappa *et al.*[37]	✓ Independently validated	✓ Consecutive cases	✓ Community controls	Not stated	✓ Yes	No	✓ Secure record	✓ Same for both groups	✓ Same for both groups	7

^a^Risk of bias was assessed using the Newcastle-Ottawa Scale [39]; ^b^ A higher overall score (maximum = 9) corresponds to a lower risk of bias.

✓ The study met this assessment criterion. ✗ This study could not be assessed with this scale. There was no unexposed cohort; both cohorts received screening. The two groups differed in terms of their age at exposure.

### What is the effect of cervical cancer screening on incidence of and mortality from invasive cervical cancer?

A summary of the evidence available to answer the question about the effect of screening on cervical cancer mortality and incidence is presented in Table 3.

One large cluster randomized trial provided cervical cancer mortality and incidence outcomes for women with a single lifetime screen compared with women with no screening history [20]. Fifty-two villages in rural India, with a total of 131,746 healthy women ages 30 to 59 were randomly assigned to one of four groups. Women in these groups were offered a single screening by HPV test, cytology or visual inspection by acetic acid, or were told how to seek screening at local hospitals. Eight-year follow-up data showed the risk of dying from cervical cancer was 35% lower among women invited to screening with HPV or cytology testing than among women not offered screening (risk ratio (RR) 0.65; 95% CI 0.47, 0.90; *P* = 0.01). Likewise, women offered screening with one of these two tests had a 44% lower risk of being diagnosed with advanced cervical cancer (International Federation of Gynecology and Obstetrics (FIGO) stage II+) than women in the control group (relative risk (rr) 0.56; 95% CI 0.42, 0.75; *P* = 0.0001). However, screening by a single lifetime HPV or cytology test did not influence overall cervical cancer (FIGO stage I+) incidence (rr 1.12; 95% CI 0.91, 1.39; *P* = 0.28). The higher risk among screened women is explained by the detection of disease in the screened groups and the fact that this was the first cervical screening procedure almost all of the women had ever undergone.

**Figure 2:**
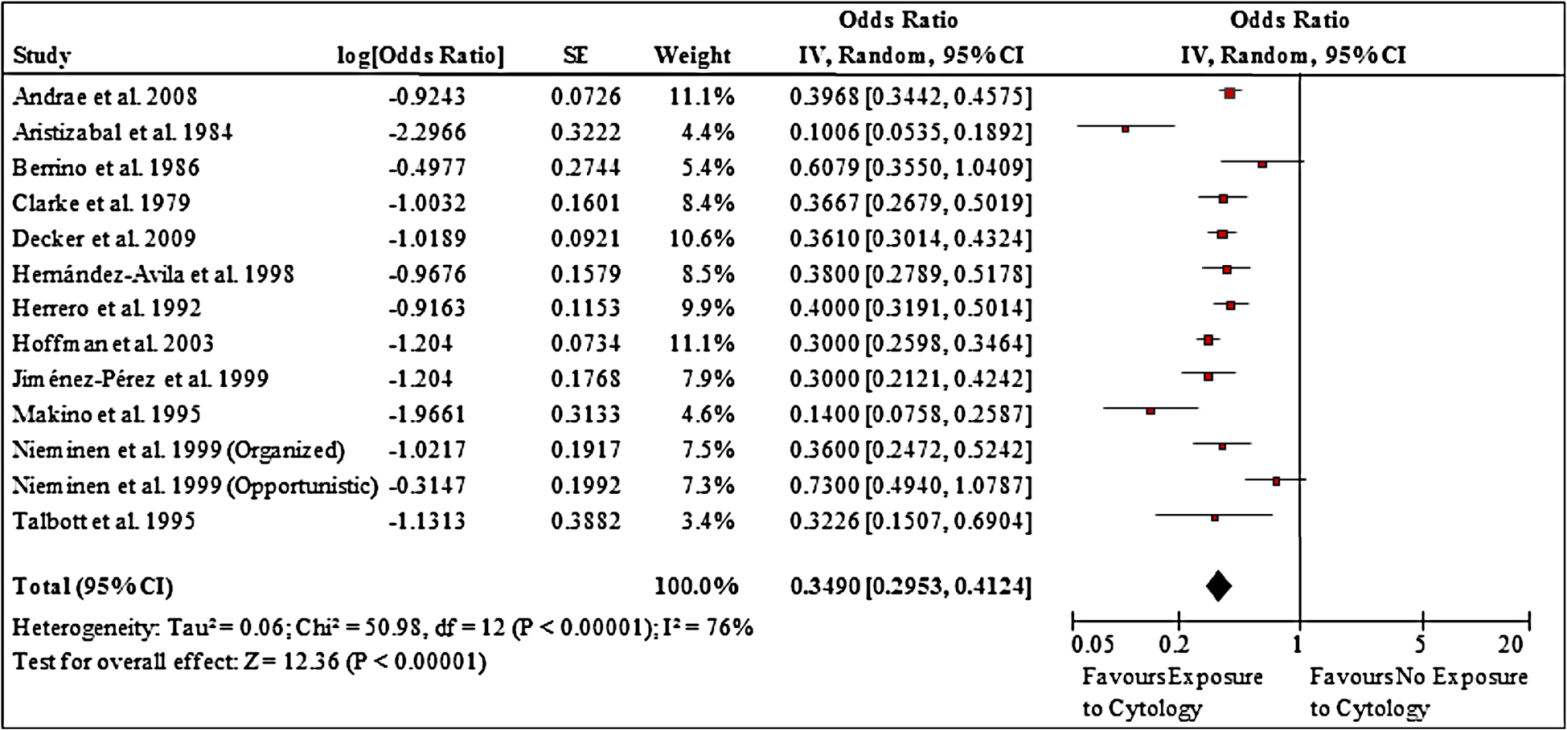
Forest plot of the effect of screening on incidence of invasive cervical cancer - exposure to cytology screening.

Observational studies conducted in nations or regions where organized screening programs are in place and/or in countries where women are likely to participate in recurrent opportunistic screening have shown significant protective effects of cytology screening. A UK-based cohort study of 116,022 women aged 25 to 69 years demonstrated the incidence of invasive cervical cancer (FIGO stage I+) was significantly lower among women who participated in the country’s comprehensive screening program (that is, they had at least one cytology test in the preceding 6 to 66 months) than among women not screened during this interval (rr 0.38; 95% CI 0.23, 0.63; *P* = 0.0002) [21]. Benefits were also apparent in a dozen case–control studies that examined exposure to cervical screening among women with invasive cervical cancer and age-matched women without the disease [15-18,23-30]. Meta-analysis of the 12 studies, which included almost 4,800 cases and 18,000 controls, showed lower odds of having undergone screening with cytology among women who were diagnosed with cervical cancer (odds ratio (OR) 0.35; 95% CI 0.30, 0.41, *P* <0.00001) (Figure 2). Despite similarities in point estimates, overlapping CIs and consistency in the direction of effect across studies, the pooled result should be applied with caution given that heterogeneity statistics were significant (Chi^2^ = 50.98, degrees of freedom (df) = 12, *P* <0.00001; I^2^ = 76%). A number of sensitivity analyses were conducted to attempt to explain the variation. The test for subgroup differences between studies conducted in more generalizable (to the Canadian context) countries versus less generalizable countries showed moderate heterogeneity (Chi^2^ = 2.27, df = 1, *P* = 0.13, I^2^ = 55.9%). All other sensitivity analyses showed minimal to no heterogeneity (Canada and US versus other countries: Chi^2^ = 0.14, df = 1, *P* = 0.71, I^2^ = 0%; mass or organized screening versus spontaneous or opportunistic screening: Chi^2^ = 0.56, df = 1, *P* = 0.46, I^2^ = 0%; any lifetime screening exposure versus exposure in previous 3 to 6 years: Chi^2^ = 0.12, df = 1, *P* = 0.73, I^2^ = 0%; self-reported screening history versus verified records: Chi^2^ = 1.28, df = 1, *P* = 0.26, I^2^ = 21.6%; case diagnosis date 1990s/2000s versus pre-1990s: Chi^2^ = 0.14, df = 1, *P* = 0.71, I^2^ = 0%; community controls versus hospital controls: Chi^2^ = 0.35, df = 1, *P* = 0.56, I^2^ = 0%).

### How does varying the screening interval affect incidence of and mortality from invasive cervical cancer?

For the question regarding the optimal frequency of screening, the search located 14 studies which included 12 case–control studies that looked at exposure to cervical screening and two cohort studies that reported incidence rates for invasive cervical cancer [16,18,19,21,22,24,27-29,31-33],[35-38]. We found no studies that reported cervical mortality outcomes related to screening intervals. Methodological variations (for example, in interval durations, interval groupings, diagnostic test exclusion periods) prevented pooling data from these studies; thus this review is unable to provide definitive answers on how often to screen. However, the evidence does offer some indications that are useful for decision-making. First, the shortest screening interval considered within each study consistently offered the greatest protective effects (for example, <1 year OR 0.14 (no CI given) [16]; <1 year OR 0.18 (95% CI 0.09, 0.35) [32]; 1 year OR 0.09 (95% CI 0.06, 0.16) [29]; <3 years OR 0.12 (95% CI 0.07, 0.20) [19]; <3 years OR 0.25 (95% CI 0.15, 0.42) [37]). Second, screening intervals of five years or less appeared to offer women substantial protection against invasive cervical cancer (for example, <5 years OR 0.3 (95% CI 0.2, 0.4) [27]; 1 to 5 years OR 0.2 (95% CI 0.1, 0.5) [28]; 1 to 3 years OR 0.27 (95% CI 0.13, 0.56) [38]; 2 years OR 0.17 (95% CI 0.08, 0.34) [29]). Third, the protective effect of screening diminished with longer intervals between tests but even intervals of 10 to 15 years showed significant protective benefits (for example, 10 to 14 year interval OR 0.4 (95% CI 0.3, 0.5); ≥15 year interval OR 0.5 (95% CI 0.4, 0.7) [27]). Finally, regardless of the specific interval, any lifetime screening was better than no history of screening (for example, interval >5.5 years compared to never screened RR 0.34 (95% CI 0.14, 0.82) [21]; interval ≥6 years compared to never screened OR 0.56 (95% CI 0.38, 0.82) [37]).

### How does varying the age at which screening is started or stopped reduce incidence of and mortality from invasive cervical cancer?

For the question on optimal ages to start and stop screening, the search located four studies including three case–control studies that examined exposure to screening among different age groups and one age comparison within a cohort study that reported incidence rates for invasive cervical cancer [22,24,27,32-34]. No studies were found that looked specifically at the age to stop screening; only one study investigated the protective benefit of screening among older women [24]. We found no studies that reported cervical cancer mortality outcomes related to age and screening history. Methodological variations across studies (for example, in overall age ranges, age groupings used for analysis) prevented pooling the data. Given the available evidence we were not able to definitively answer the question regarding ages to initiate and discontinue cervical screening, however we were able to draw a few themes from the data. Participation trends showed very high screening attendance among young women, high attendance among middle-aged women, and consistently lower participation in older groups of women [32,33]. Despite very high participation among younger women, the benefit of screening below age 30 is unclear. Significant benefits were found in one study (OR 0.42; 95% CI 0.24, 0.74) [24], non-significant benefits were observed in another study (OR 0.7; 95% CI 0.3, 2.1) [27], and no benefits were found in a third study (screened at ages 20 to 21 OR 1.51; 95% CI 0.95, 2.38; screened at ages 22 to 24 OR 1.11; 95% CI 0.83, 1.50) [34]. Screening decisions for women under age 30 must consider the balance between potential benefits and potential harms and economic costs. Alternatively, the evidence indicates exposure to cytology screening provides a substantial protective effect in women 30 years and older (for example, screened at ages 30 to 65 OR 0.40 (95% CI 0.34, 0.47) [24]; ages 40 to 59 OR 0.3 (95% CI 0.2, 0.4) [27]; ages 42 to 44 OR 0.37 (95% CI 0.29, 0.48); ages 52 to 54 OR 0.26 (95% CI 0.19, 0.36) [34]) and there is some evidence this protective effect remains strong in women over 65 years (OR 0.36; 95% CI 0.24, 0.53) [24].

## Discussion and conclusions

The ultimate goal of cervical screening is to decrease the incidence of and subsequent mortality from invasive cervical cancer. The available evidence supports the conclusion that screening does offer protective benefits and is associated with a reduction in these critical outcomes. An RCT in India showed that even a single lifetime screening test significantly decreased mortality from and incidence of advanced cervical cancer compared to no screening. Cytology screening was shown to be beneficial in a cohort study that found testing significantly reduced the incidence of invasive cervical cancer compared to no screening. Pooled evidence from a dozen case–control studies also indicated a significant protective effect of cytology screening. This review found no conclusive evidence for establishing optimal ages to start and stop cervical screening, or to determine how often to screen; however the available data suggests substantial protective effects for screening women 30 years and older and for intervals of up to five years.

### Limitations

The findings are impacted by the biases and limitations of the literature and the included studies. For the question on the effect of cervical screening on the outcome of mortality, all of the data came from one RCT of a single lifetime screen offered to women in rural villages in India with follow-up limited to eight years. The bulk of the evidence used to assess the effect of screening on the incidence of invasive cervical cancer and to address the questions about optimal screening intervals and ages was taken from low and very low GRADE quality observational studies, the results of which need to be considered with caution. Further, aside from the location of the studies offering some explanation, the large amount of heterogeneity between studies included in the meta-analysis cannot be accounted for by the factors explored in the sensitivity analyses; consequently the pooled estimate should also be applied with caution. It is also important to acknowledge the potential for observational studies to present additional risks that could introduce bias in favor of screening including: earlier diagnosis in screen-detected cases (lead bias), over-representation of women with a lengthy pre-clinical stage (length bias), and over-representation of healthier participants among those who attend for screening (volunteer bias). Finally, we restricted our search to papers in English or French, thus we may have missed relevant data in papers written in other languages.

### Implications for further research

Although this review identified research evidence that supports the practice of cervical cancer screening, there remain unanswered questions, particularly about newer HPV technologies. Compared to studies that focus on outcomes after cytology screening, the evidence base concerning the relative effectiveness of HPV screening is limited. While minimal evidence was found for cytological testing, no studies were found that looked at optimal screening intervals or ages to commence or discontinue HPV testing that also met the inclusion criteria (for example, included outcomes of invasive cancer incidence and mortality). More evidence is needed on the harms of HPV testing (for example. false positive rates) and the related potential for unnecessary, and possibly harmful, diagnostic and treatment procedures. The HPV FOCAL Trial, currently underway in British Columbia, is one study that may provide answers to some of these important questions [43].

## Abbreviations

df: Degrees of freedom; FIGO: International Federation of Gynecology and Obstetrics; GRADE: Grading of Recommendations Assessment, Development and Evaluation; HPV: Human papillomavirus; OR: Odds ratio; RCT: Randomized controlled trial; rr: Relative risk; RR: Risk ratio; USPSTF: United States Preventive Services Task Force.

## Competing interests

The authors declare that they have no competing interests.

## Authors’ contributions

All authors performed tasks involved in conducting the full systematic review. LP drafted the initial version of the manuscript. All authors reviewed, contributed revisions and approved the final manuscript prior to submission.

## Note

This paper is based on a full report conducted by the McMaster Evidence Review and Synthesis Centre entitled *Screening for Cervical Cancer* which can be found at http://canadiantaskforce.ca/wp-content/uploads/2013/01/Systematic-Review-Cervical-Cancer.pdf?9d7bd4.
